# A Defined Patho-Mechanism for Acute Radiation Syndrome Death and a Three-Drug Regimen to Prevent It

**DOI:** 10.3390/ijms27062659

**Published:** 2026-03-14

**Authors:** William E. Fahl, Hannah R. Goesch, Sarah R. Goesch, Bryan L. Fahl

**Affiliations:** 1 Obvia Pharmaceuticals Ltd., 319 Bernardo Avenue, Mountain View, CA 94043, USA; 2 McArdle Laboratory, Wisconsin Institutes for Medical Research, University of Wisconsin-Madison, Madison, WI 53705, USA

**Keywords:** ROS scavenger, jejunal toxicity, nuclear, sepsis, GCSF

## Abstract

Death from acute radiation syndrome (ARS) has been a long-standing threat. Given the current heightened risk of a nuclear event, e.g., a conflict bomb, terror bomb, reactor core dispersion, or recurrent exposure to medical radiation, a systemic treatment to reduce or eliminate ARS death would be beneficial. This study utilizes step-wise progression to (i) identify why lethally irradiated mice die from ARS and (ii) identify a multidrug regimen, administered before or after irradiation, that prevents or treats ARS pathologies to significantly suppress or eliminate ARS death. Lethal blood-borne *E. coli* septic infection was found in 97% of near-death, irradiated mice; this observation was consistent with the numerous breaches observed in GI histology showing a broken and breached GI epithelium and GI muscularis externa. Our study found (i) a new and clear explanation of why irradiated mice die from ARS; (ii) identified two drugs (PrC-210, ciprofloxacin), which, when administered minutes pre-radiation, conferred 100% survival benefit or 56% when administered a day after irradiation; and (iii) a three-drug regimen (PrC-210, ciprofloxacin, GCSF) that conferred 92% survival benefit when administered 1–2 days after radiation. These drug regimens can be “field-deployed” to field staging areas and home medicine chests to enable the simple, widespread use of the regimens in the face of radiation threat.

## 1. Introduction

Burns associated with radiation exposure were first described by Tesla after he exposed his hand to X-rays in 1896 [1], but systemic illness and organ failure following radiation exposure were not described in detail until after the Hiroshima and Nagasaki bombings in August 1945 [2,3]. A Japanese woman, Midori Naka, who was present in Hiroshima, was the first human in 1945 whose death was attributed to what was then referred to as “Atomic bomb disease” [4]. In the 80 years since, this widespread set of human post-radiation organ toxicities has been known as radiation sickness and more recently as acute radiation syndrome (ARS) [5]. The panoply of organ failure seen after exposure to a lethal ionizing radiation dose has been described following many human exposures to radiation, including Hiroshima/Nagasaki, i.e., through the Atomic Bomb Casualty Commission [5], Chernobyl [6], and Fukushima [7], as well as more limited examples of employee exposures to lethal or sublethal radiation doses [8,9].

The organ failure that typically precedes ARS death is principally seen in bone marrow and gastrointestinal sites at radiation doses of 1–9 Gy [10,11], which, with successful pre- or post-radiation treatment, could be survived. Neurovascular failure in ARS is typically seen at far higher radiation doses (50 Gy) that are not survivable. In humans, the gastrointestinal epithelium is normally replaced every 3–5 days, so the radiation-induced apoptosis of GI epithelial stem cells can lead to significant denuded tissue sites. The radiation-induced death of bone marrow stem cell populations leaves humans with little ability to address any infectious agent post-radiation.

The human risk of radiation exposure, possibly catastrophic in proportion, has steadily risen and has never been as high as it is today [12]. Multiple world events, including the shelling of nuclear power plants in Ukraine [13] and the U.S. announcement to resume nuclear weapons testing after a 33-year hiatus [14], constitute a clear escalation in nuclear risk. Therefore, the need for a field-ready drug regimen that is able to protect civilian and military personnel, before and especially after any radiation event, has never been greater.

A substantial U.S. government infrastructure has been assembled over the past decades [15,16] to study and strategize responses to enable survivor support following any event from accidental radiation spills to a no-warning nuclear attack from international aggressors [12].

The first example of systemically administered “radioprotection” was seen in 1949 following the systemic administration of the thiol-containing amino acid cysteine to mice immediately before whole-body irradiation [17]. Countless strategies have since been pursued world-wide in an attempt to identify both pre-radiation-administered “radioprotectors” [18,19] and post-radiation-administered “radiomitigators” [20,21]. The list is long and the attempts expensive, with little or no success.

Our goal in this study, rather than to again describe a series of toxicities observed in mammals exposed to lethal radiation, was to identify the cause of death in lethally irradiated mammals, here outbred mice, and, by so doing, enable a targeted therapeutic regimen that specifically addresses, or ideally suppresses to background, the identified ARS death patho-mechanism. Our goal was to create a likely multidrug regimen that is “field-deployable,” wherein the “field” represents ***both*** high-risk military conflict sites and also home medicine chests, where at-risk soldiers and civilians can administer a regimen themselves, either (i) minutes before or (ii), if they survive the thermoblast, a day or days after a nuclear event, to achieve up to 100% “radioprotection” (administered before) or the best possible (up to 92%) “radiomitigation,” when administered after irradiation. Analyses of digital histologic images of gastrointestinal damage following the lethal whole-body irradiation of mice taken in our earlier study [22] led us to a hypothesis that considered widespread septic infections in irradiated animals. The pursuit of this hypothesis resulted in (i) a confirmed ARS death patho-mechanism; (ii) a two-drug regimen, taken minutes before irradiation, that confers a 100% survival benefit; and (iii) a three-drug regimen, taken 24 h after irradiation, that confers a 92% survival benefit to mice administered an otherwise 96% lethal dose of whole-body radiation.

## 2. Results

### 2.1. Mouse Irradiation, ROS Production and GI Degradation

**Figure 1:**
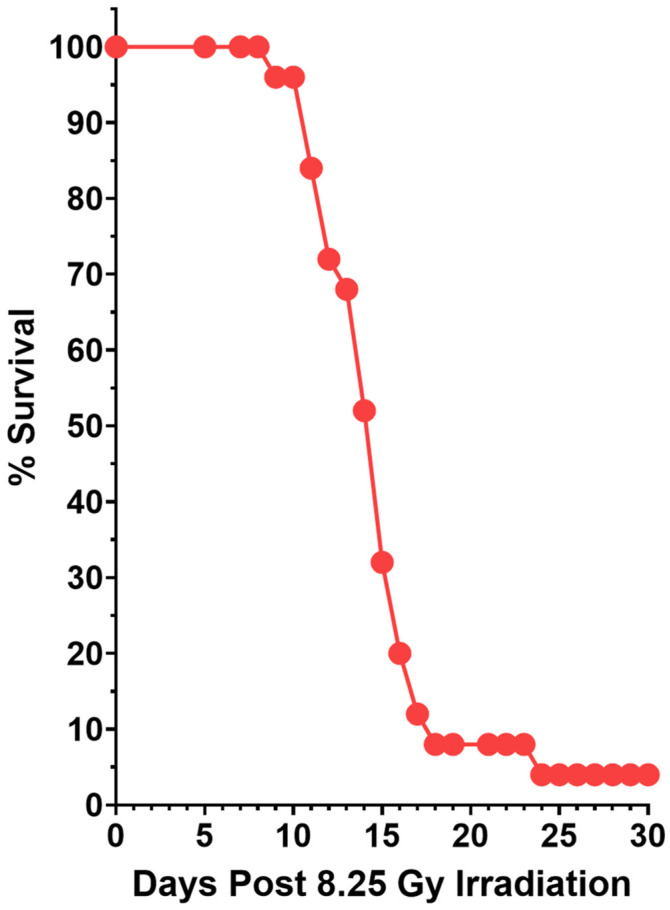
Post-irradiation death following acute radiation syndrome. Twenty-five female outbred ICR mice were irradiated with an 8.25 Gy dose of 320 keV X-rays. Mice were observed over the next 30 days, and deaths were recorded.

We irradiated 25 outbred ICR mice with 8.25 Gy of 320 keV X-rays; the mice were returned to cages and observed over the next 30 days (Figure 1). By observation, the mice developed external signs of acute radiation syndrome, which included (i) a hunched posture, (ii) the absence of movement in a setting with cage-mates, (iii) a ragged coat appearance, (iv) being cold to touch when picked up, and (v) death. The 8.25 Gy dose of X-rays was lethal to 96% of the irradiated mice in the following 24 days.

**Figure 2:**
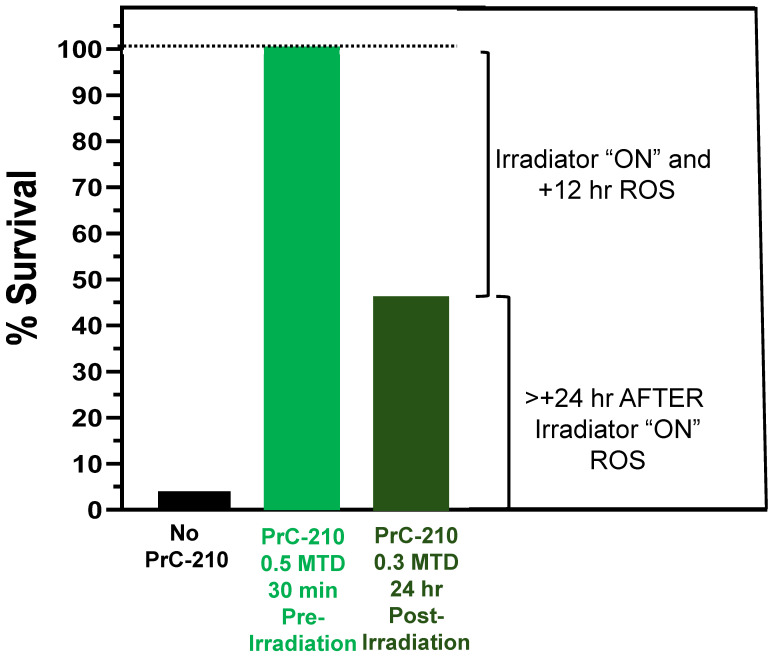
The PrC-210 suppression or mitigation of radiation-induced death in ICR mice. Single systemic doses of PrC-210 were administered either (i) IP (0.5 MTD, 262 mg/kg, 30 min before an LD_100_ dose of Cs^137^ radiation) [22] or (ii) IP (0.3 MTD, 157 mg/kg, 24 h after an LD_96_ dose of 320 keV X-rays) [23]. Mice were observed over the next 30 days, and deaths were recorded. Data are used from indicated references with publisher permission.

In Figure 2, we see that single systemic doses of the PrC-210 aminothiol ROS scavenger conferred: (i) 100% survival against an otherwise 100% lethal dose of Cs^137^ gamma irradiation when administered IP (intraperitoneally) 15–30 min before irradiation [22] or (ii) a 44% survival benefit against a 96% lethal dose of X-ray gamma irradiation when administered IP 24 h after irradiation [23]. In ongoing experiments, using an intravenous injection (8.25 Gy) of a coelenterazine derivative into irradiated mice [24] to externally image ROS production in irradiated mouse organ sites, we saw three peaks of ROS production in post-irradiation ICR mice, (i) during the irradiator ON time, (ii) at 12 h post-irradiation, and (iii) at 144 h post-irradiation. All three ROS peaks were reduced to background in mice that received systemic PrC-210 either 30 min before [peaks (i) and (ii)] or 24 h after irradiation [peak (iii)].

**Figure 3:**
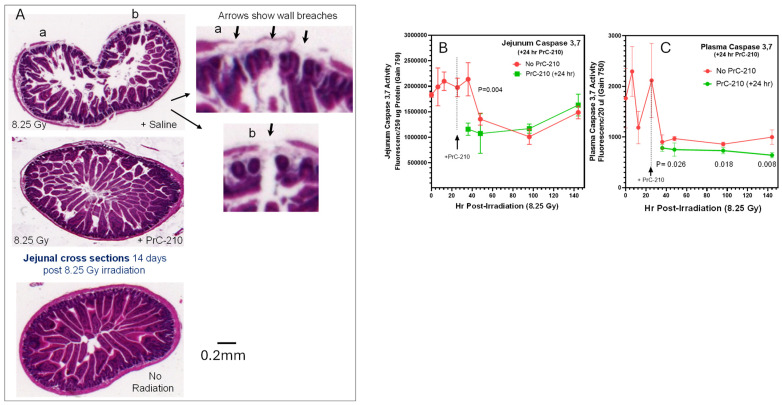
Radiation-induced cell death in mouse jejunum tissue. (**A**) A 2 cm segment of the jejunum was removed from euthanized mice (4 cm below the gastric/jejunum junction) 14 days following irradiation. H&E histology images showed radiation-induced jejunal villi and jejunal wall breeches (a,b), which were not seen in unirradiated or PrC-210-treated mice. (**B**) At the indicated post-irradiation timepoints, jejunum segments were removed, rinsed, and then homogenized. (**C**) Blood plasma was also collected from the same mice. At the indicated timepoints, the levels of the caspase 3,7 cell death biomarker were quantified in both the jejunal tissue homogenates and blood plasma recovered from the same mouse. The jejunum caspase 3,7 death marker level was markedly reduced in the jejuna of mice injected with PrC-210 24 h after irradiation (*p* = 0.004). Likewise, the blood plasma levels of caspase 3,7 were reduced following PrC-210 administration.

In the above-mentioned early ROS production images of irradiated mice, with the goal of understanding which organs experienced significant radiation-induced ROS damage following lethal irradiation, we analyzed ROS produced from sites over the gastrointestinal tract. Because of the critical roles of GI sites in water and nutrient absorption, as well as their role as a huge repository of gut bacterial flora, they were a primary site of study. Jejunal tissue segments were removed from the same site in irradiated mice on day 14 post-irradiation (see Section 4), formalin-fixed, H&E-stained, and studied by white light microscopy (Figure 3A). The observations of jejunal histology in these mice included the following: (i) (bottom panel) in unirradiated ICR mice, jejunal villi, lamina propria, and muscularis externa were intact and normal; (ii) (top panels) in irradiated mice, all jejunal elements, including villar structure and number, lamina propria, and most importantly muscularis externa integrity and intactness, were severely degraded, and there were multiple tissue gaps in both the villi and the muscularis externa (top panel enlarged images) that would allow for the leakage of any gastrointestinal lumen contents into the mouse abdominal cavity; and (iii) (middle panel) jejuna from mice that received a single 0.3 MTD PrC-210 dose at +24 h post-irradiation contained largely normal villar structure and density and ***no*** detectable gaps or breaks in the muscularis externa capsid wall. Though these images are from jejunal samples, colon histology samples from the same mice showed similar conclusions. The quantitation of both radiation-induced jejunal damage and the PrC-210-conferred suppression of jejunal radiation damage was previously reported [23]. Consistent with the histology images, when we analyzed homogenates prepared from jejunal segments from the same mice (Figure 3B), we saw a large elevation in jejunal caspase 3,7 levels in irradiated mice and the suppression of the caspase 3,7 death marker essentially to background (*p* = 0.004) in mice treated with the single PrC-210 dose. In plasma from these mice (Figure 3C), the statistically lower level of caspase 3,7 was maintained through the six days post-irradiation during which blood samples were collected.

### 2.2. Acute Radiation Syndrome Blood Sepsis and Death

**Figure 4:**
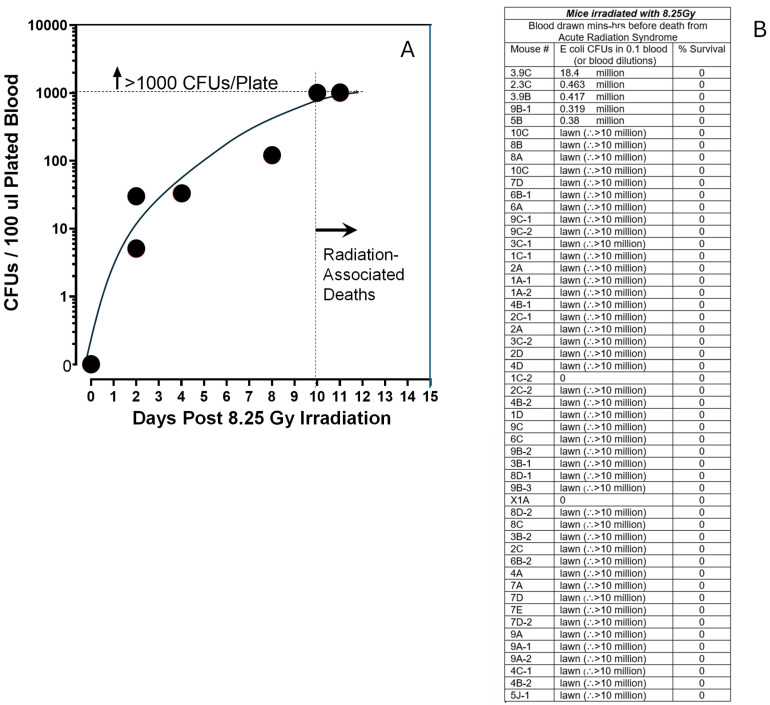
Blood-borne septic microorganism infection in irradiated mice in the days following 8.25 Gy irradiation. (**A**) A total of 100 µL of sterilely collected blood from irradiated mice on the indicated days post-irradiation was plated on blood agar plates and cultured for 24–48 h at 37 °C. Colony counts were recorded. (**B**) In a large experiment of 8.25 Gy-irradiated mice, 51 mice were euthanized at the point where they were observed to be within 1–3 h of death from acute radiation syndrome, typically 10–13 days post-irradiation (i.e., hunched posture, lack of motion in cage, coat was ragged and unsmooth, body was cold to touch). Sterilely collected blood (100 µL) was collected and plated on blood agar; in some cases, blood dilutions were also plated to better assess actual colony numbers in 100 µL of blood.

After seeing the presence of breaches in the muscularis externa GI capsid in the irradiated mice, we reasoned that this could provide both (i) a pathway for the escape of GI floral bacteria and (ii) a patho-mechanism for explaining deaths in acute radiation syndrome by septic infection. To test this hypothesis, we irradiated a large group of ICR mice and then euthanized them and cultured sterilely drawn blood from the mice at timepoints after they received a 96% lethal dose of whole-body radiation. As shown in Figure 4A, we were startled to find blood-borne microorganism CFUs in 100 µL of individual mouse bloods as early as 2 days after the 8.25 Gy whole-body irradiation. CFU levels in bloods rose exponentially in mice in the first 10 days post-irradiation during which we drew the bloods, basically achieving uncountable “lawns” on blood agar plates by 9–10 days following irradiation. Notably, the first mice died on days 9–10 following irradiation from observable acute radiation syndrome (Figure 1). Over the course of the three following experiments, each including 8.25 Gy irradiation of untreated ICR mice, 51 mice were identified in our twice-daily cage inspections to be within mins-hrs of death from apparent acute radiation syndrome (Figure 4B). Sterile blood was collected from each mouse, and 100 µL of blood or 100 µL of a blood serial dilution was spread on blood agar plates. In 49 of 51 mice, we observed either CFU lawns or countable CFUs in serially diluted blood. Serial dilutions, generally out to 1 million-fold, yielded CFU/mL mouse blood values in the near-death mice of >10 million CFUs/mL. Two near-death mice, 1C-2 and X1A, contained zero CFUs in 100 µL of their blood.

**Figure 5:**
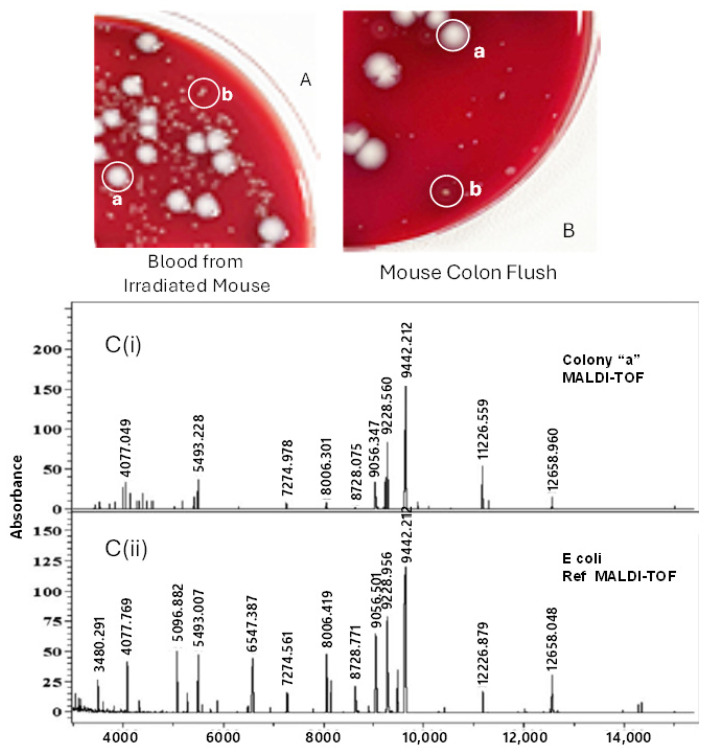
The determination of blood microorganism identity and possible origin in radiation-compromised mouse GI tissue. (**A**) Blood from an irradiated, near-death (day + 12) mouse was sterilely collected, serially diluted with PBS, and plated on blood agar plates. After 24–48 h at 37 °C, two different colony types were clearly visible, “a” and “b.” (**B**) The colon from an unirradiated ICR mouse was sterilely removed, and its contents were flushed into a sterile tube using 1 mL of sterile PBS. Serial dilutions of the colon flush were plated on blood agar plates and photographed after 48 h at 37 °C. (**C**(i),**C**(ii)) Individual colonies (“a” and “b” from both source plates) were expanded in Luria broth and delivered to a clinical laboratory setting where protein extracts from the expanded colonies were analyzed by MALDI-TOF mass spectrometry, and the mass spectrograms were then compared to their library of known bacteria. In the example shown here, the “a” colony mass spectrogram is identical to the library *E. coli* reference mass spectrogram. A similar approach was used to identify the “b” colony as *Lactobacillus suis*.

Only two colony morphologies, types “a” and “b”, were ever observed in the plated bloods from irradiated mice (Figure 5A), and in >99% of cases, only type “a” colonies were seen. If our nascent hypothesis of blood sepsis was correct, then we would expect to find these same colony-type organisms in the samplings of normal ICR mouse GI flora. To test this, we sterilely dissected segments of both the jejuna and colon from an unirradiated ICR mouse, flushed the luminal contents with sterile PBS into a tube and then plated dilutions of the GI floral microorganisms on blood agar plates (Figure 5B). The colony morphologies in the GI flush samples, to the eye, were identical to the colony morphologies seen in the irradiated mouse bloods. To first identify the organisms, and second determine that irradiated blood and GI flush organisms were the same, a type “a” colony and a type “b” colony from both irradiated blood and GI flush were picked and expanded overnight in Luria broth. Bacterial protein extracts from these bacteria were then prepared and analyzed by MALDI-TOF mass spectrometry in a clinical laboratory setting (https://www.slh.wisc.edu/). Figure 5(C(i),C(ii)) show an example in which an experimental colony “a” extract MALDI-TOF mass spectrogram is found to be identical to a library MALDI-TOF mass spectrogram from *E. coli*. A similar process was used to determine the identity of colony “b” as *Lactobacillus suis*. The final analysis identified *E coli* and *Lactobacillus suis* in GI flushes and the same organisms found in every CFU-positive blood sample taken from near-death, irradiated mice.

**Figure 6:**
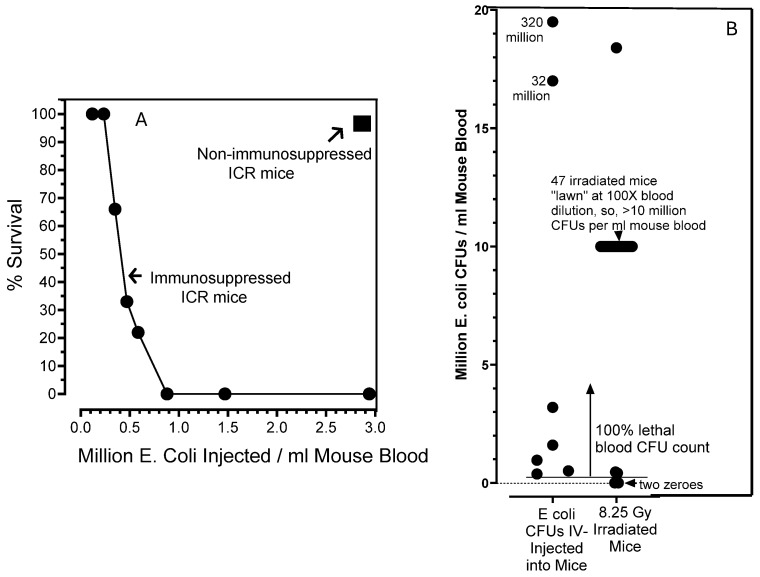
The determination of a lethal septic *E. coli* load when injected IV into healthy, immunocompromised ICR mice. (**A**) Colony “a” *E. coli* cells, from an irradiated mouse blood sample grown on a blood agar plate, were grown in an overnight Luria broth culture at 37 °C. Serial dilutions of the overnight culture were titrated on blood agar plates; the overnight culture was found to contain 1.4 × 10^9^ *E. coli* cells per ml. Aliquots of the overnight culture were diluted in sterile tubes with sterile PBS to yield bacterial suspensions containing 0.3 to 320 million *E. coli* CFUs per 150 µL. ICR mice which had been immunocompromised (or not) in the week before tail vein *E. coli* injections (see Section 4) were individually immobilized in an injection holder, and tail veins were dilated in warm water and then injected with 150 µL aliquots containing known numbers of *E. coli* cells. At least three mice were in each group; injected mice were returned to cages. Mouse survivals in the days following *E. coli* IV injections are shown. (**B**) Blood-borne *E. coli* CFUs per ml of blood in tail vein-injected and 51 irradiated mice are plotted. A total of 49 out of 51 irradiated mouse bloods analyzed contained *E. coli* CFU numbers that were above the minimal “lethal” CFU number determined in the Panel A experiment.

To determine whether a septic, blood-borne *E. coli* infection, ***alone***, could explain acute radiation syndrome deaths in irradiated mice, and if so, what blood-borne *E. coli* lode was lethal, an “a” colony was picked and expanded overnight in Luria broth. The overnight culture was then titrated by serial dilution and held at 4 °C to preclude further bacterial growth; this yielded cultures of around 1.4 billion *E. coli* cells per ml of broth. Healthy ICR mice then individually received a tail vein injection of 150 µL of PBS containing 0 to 320 million *E. coli* cells, and the mice were returned to cages and observed. In Figure 6A, we see that death occurred in immunosuppressed mice that received anywhere from 0.3 million to 320 million *E. coli* cells per ml of mouse blood. Immunosuppressed mice had received two IP injections of Cytoxan and two IP injections of prednisone (see Section 4) in the 7 days prior to the *E. coli* injection. In ICR mice that did not receive the immunosuppressive regimen, a bolus of 5.4 million *E. coli* cells (i.e., 3.0 million per ml blood) caused neither death nor discernible illness. In the plot shown in Figure 6B, we see that all 49 of the “blood CFU-positive” irradiated mice (see Figure 4) contained blood-borne *E. coli* loads that exceeded the minimal number of *E. coli* shown to inflict death, when *E. coli*, ***alone***, were injected into healthy, immunosuppressed mice.

**Figure 7:**
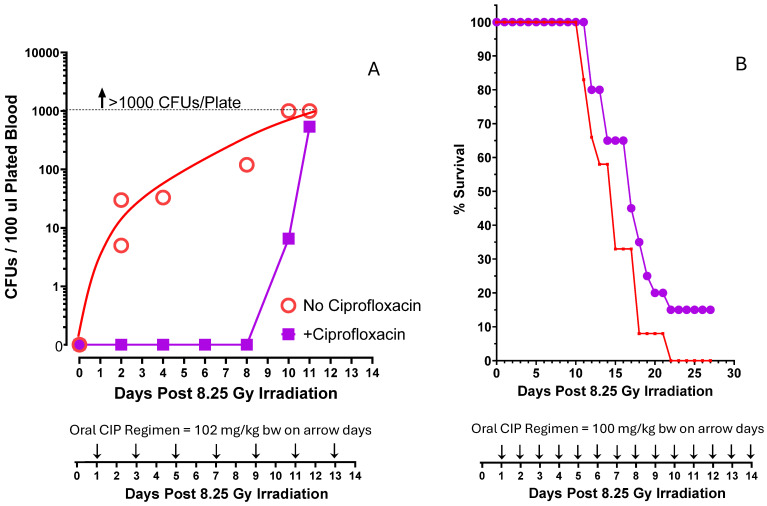
The ciprofloxacin treatment of irradiated mice. ICR mice were all irradiated with 8.25 Gy. (**A**) Here irradiated mice received an oral gavage dose of ciprofloxacin (172 mg/kg bw) every other day following irradiation. Whereas mouse blood bacterial CFUs were detectable shortly after irradiation in non-ciprofloxacin-treated mice (plot taken from Figure 4A), mice that were irradiated but also received alternate-day oral ciprofloxacin showed no detectable blood bacterial CFUs through day 8 post-irradiation. After day 8, CFUs rose very rapidly, and none of the mice on this ciprofloxacin dose regimen survived. (**B**) In a subsequent experiment, groups of 20 mice all received 8.25 Gy irradiation; 20 then received no treatment, and 20 received *daily* oral gavage ciprofloxacin (100 mg/kg) for 14 days following irradiation. With this ciprofloxacin regimen, there was a 15% survival benefit over 30 days.

In the first experiment to determine whether the observed radiation-induced *E. coli* sepsis could be pharmacologically suppressed, we administered an oral gavage dose of ciprofloxacin *every other day* to 8.25 Gy-irradiated mice (Figure 7A) and asked whether it affected the presence of blood-borne *E. coli* in these mice versus irradiated mice that received no ciprofloxacin. For the first eight days following irradiation, there was no discernible *E. coli* in mouse bloods from ciprofloxacin mice versus the exponential increases that were seen in irradiated mice untreated with antibiotic (Figure 7A). However, by day +11 post-irradiation, the blood levels of *E. coli* had reached those seen in non-antibiotic-treated mice, and in the end, the 30-day survival rate in both groups of mice shown in Figure 7A was the same, 0–5%. In the next experiment, we changed the ciprofloxacin regimen to now include *daily* ciprofloxacin doses at 100 mg/kg versus the 102 mg/kg *every other day* used in Panel A (Figure 7A vs. Figure 7B). When these groups of 20 irradiated mice were carried through 30 days post-irradiation, a 15% survival benefit was observed in the ciprofloxacin mice (Figure 7B).

### 2.3. A Multidrug Regimen to Suppress Acute Radiation Syndrome Death

**Figure 8:**
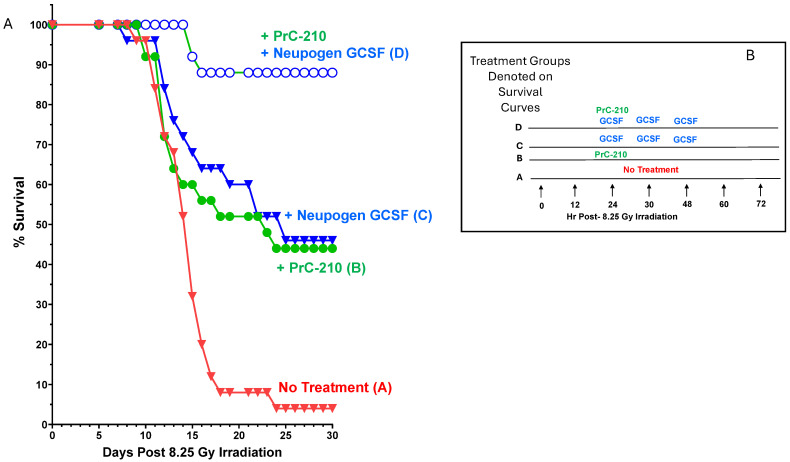
PrC-210- and GCSF-conferred post-irradiation mouse survival. Survival in four groups (25 ICR mice per group), all of which received 8.25 Gy of whole-body irradiation. (**A**) Mice were observed twice daily for survival for the 30 days following the 8.25 Gy irradiation. (**B**) A schematic illustrating the post-irradiation times at which (i) a single IP PrC-210 dose (0.3 IP MTD, 157 mg/kg bw) or (ii) three SC doses of GCSF (Neupogen, Amgen) or (iii) both drugs were administered to irradiated mice, starting 24 h after irradiation.

In an earlier study from our lab [23], several combined regimens of IP PrC-210 and SC GCSF (Neupogen) were tested to determine what combined survival benefit could be achieved with the two drugs administered beginning at +24 h post-irradiation. In Figure 8, we see that the two drugs were almost perfectly additive in their survival benefits when dosed in the regimen shown in Figure 8B. The individual survival benefits of 43% (PrC-210) and 45% (GCSF) achieved a profound survival benefit of 88% when administered by their separate delivery routes into groups of 25 irradiated mice.

The present-day needs for either a “prevention” regimen against subsequent irradiation or a “treatment” regimen against prior irradiation places a profound emphasis on: (i) a “field-ready” drug regimen that could be available through government/military stockpiles as well as “main street” pharmacies to civilian populations and (ii) a multidrug regimen that will confer the greatest survival benefit to at-risk military and civilian populations. To address both needs (i) and (ii), we undertook a series of radiation survival experiments using multidrug regimens in both “prevention” and “treatment” protocols to identify the best possible field-ready options available to at-risk military and civilian populations.

**Figure 9:**
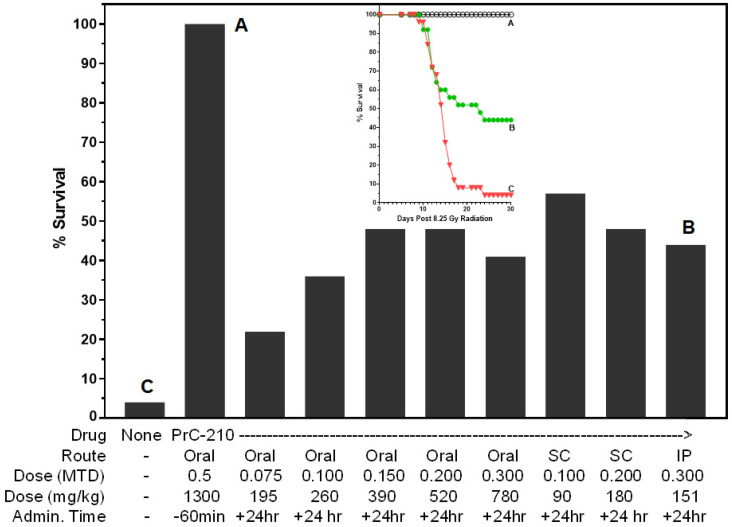
PrC-210-conferred pre-irradiation and post-irradiation mouse survival. Thirty-day survival in mouse groups (25 ICR mice per group), all of which received 8.25 Gy of whole-body irradiation. Pre-irradiation PrC-210, IP (30 min before, [22]) or oral (60 min before, [25]), conferred 100% survival against an otherwise 100% lethal radiation dose. The administration of PrC-210, alone, 24 h post-irradiation, by SC injection or oral gavage also conferred significant survival benefits. Mice were observed twice daily for survival for the 30 days following the 8.25 Gy irradiation. The final survival percentages (denoted A,B,C) on inset survival curves are also shown as bar charts labeled A, B, C. This same practice of plotting final survival percentages as bar chart heights is also used in Figure 10 and Figure 11.

**Figure 10:**
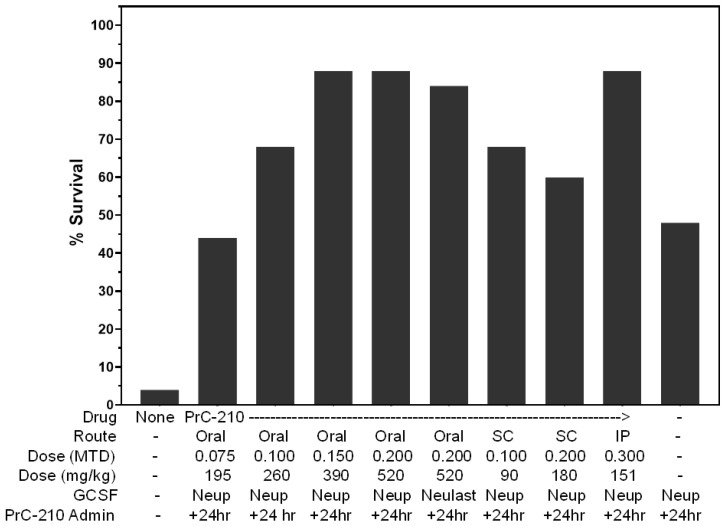
PrC-210- and GCSF-conferred post-irradiation mouse survival. Thirty-day survival in mouse groups (25 ICR mice per group), all of which received 8.25 Gy of whole-body irradiation. Pre-irradiation PrC-210, IP (30 min before [22]) or oral (60 min before [25]), conferred 100% survival against an otherwise 100% lethal radiation dose. The administration of PrC-210 alone 24 h post-irradiation, by SC injection or oral gavage, conferred significant survival benefits. Mice were observed twice daily for survival for the 30 days following the 8.25 Gy irradiation.

**Figure 11:**
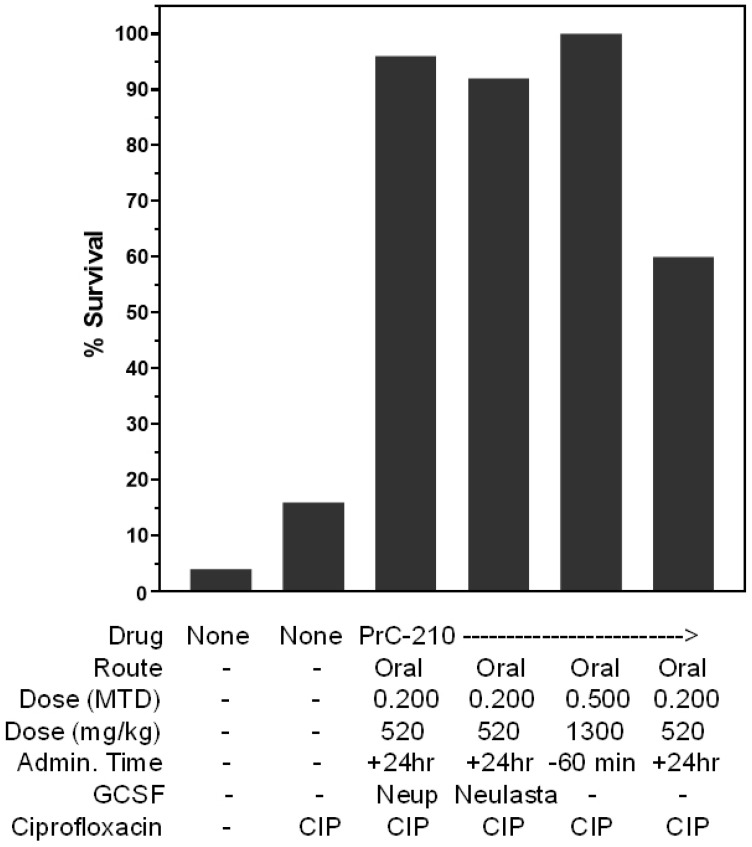
Combined PrC-210-, GCSF- and ciprofloxacin-conferred post-irradiation mouse survival. Thirty-day survival in mouse groups (25 ICR mice per group), all of which received 8.25 Gy of whole-body irradiation. The pre-irradiation oral PrC-210 plus oral ciprofloxacin regimen, i.e., the “DuoProtect^2®^ Card, conferred 100% survival against an otherwise 96% lethal radiation dose. Oral PrC-210 plus oral ciprofloxacin and SC GCSF (Neupogen) or pegylated GCSF (Neulasta) conferred 92% survival benefit. Mice were observed twice daily for survival for the 30 days following the 8.25 Gy irradiation.

In the first set of experiments, we sought to optimize the optimum dose of PrC-210 when administered to irradiated mice in a “field-ready” oral dose or a less “field-ready” SC dose (Figure 9) in both “prevention” and “treatment” settings. As shown in Figure 9, Figure 10 and Figure 11, we conducted full 30-day survival studies for mice treated as indicated beneath the individual bars, and for concise comparison, the final survival values at +30 days are presented here as simple bars (see Survival Plot insert in Figure 9). The mouse groups each contained an N of 25. In Figure 9, we see the 100% survival of mice that received an oral 0.5 MTD (900 mg/kg, this oral MTD dose is from 2010; it was done in “fasted mice”) PrC-210 dose 60 min before a 100% lethal radiation dose. We then tested a range of oral PrC-210 doses (0.075–0.3 MTD, i.e., 195–780 mg/kg, a new 2025 oral MTD of 2600 mg/kg for non-fasted mice) administered once at +24 h post-irradiation. The +24 h oral PrC-210 dose (0.150–0.200 MTD, 390–520 mg/kg) that conferred the maximum survival benefit of 44% (i.e., 48–4%) compared well with the survival benefits conferred by the optimum SC dose (52%, 0.1 MTD) and the optimum IP dose (43%, 0.3 MTD) shown in Figure 9.

When the optimum oral PrC-210 dose (0.20 MTD, 520 mg/kg), administered once at +24 h, was combined with the optimum three-dose SC GCSF (Neupogen) regimen, the survival benefit of 84% (88–4%) equaled (Figure 10) the previously observed [23] 84% survival benefit seen for IP PrC-210 + GCSF (Neupogen), also shown in Figure 10. The combination of the optimum SC PrC-210 dose (0.10 MTD) with the optimum three-dose SC GCSF (Neupogen) regimen also yielded a high survival benefit of 68%. The substitution of a single injection of pegylated GCSF (Neulasta) at +24 h for the three-injection Neupogen regimen complemented by the single oral PrC-210 dose (0.20 MTD) also yielded a very high survival benefit of 80%.

In light of the massive *E. coli* sepsis found in these irradiated mice (Figure 4), and with a 15% survival benefit conferred by daily ciprofloxacin, alone, post-irradiation (Figure 7B), in the final set of irradiation experiments, we assembled a three-drug “treatment” regimen (Figure 11) in which daily oral ciprofloxacin was included with the optimum single oral PrC-210 dose (0.20 MTD) and either three-dose Neupogen or one-dose Neulasta regimen, and the outcome basically showed an additional additive survival effect in which the small ciprofloxacin benefit was added to the singular PrC-210 benefit (43%, Figure 9) and the singular Neupogen (44%) or Neulasta benefits. Notably, the administration of PrC-210 at +24 h plus ciprofloxacin also conferred an additive survival benefit (56%, i.e., 60–4%), and as expected, the administration of the single PrC-210 dose at 60 min before irradiation followed by ciprofloxacin conferred the same survival benefit (100%, Figure 11) as that seen when PrC-210 alone was administered at 60 min before irradiation (Figure 9 [25]).

## 3. Discussion

In this study, we conducted a step-by-step series of analyses (i) to identify the reason why lethally irradiated animals die at the end of the acute radiation syndrome pathology progression and, with this knowledge, (ii) to identify a multidrug regimen, administered before or after irradiation, that logically prevented or treated the individual pathologies to confer substantial, or complete, protection against ARS death. Nearly as important as the specific drug regimen was the goal to visualize a means to “field-deploy” the drug regimen elements to field staging areas and home medicine chests to enable the simple, widespread use of the regimen in the face of radioactive threat. ARS is a long-standing, 80+-year threat, and given the risk of a significant nuclear event, whether a conflict bomb, terror dirty bomb, reactor core dispersion, or exposure to low-dose/long-term medical radiation, a simple, systemic treatment to reduce or eliminate radiation risk would be beneficial.

Because ionizing radiation-induced ROS (Reactive Oxygen Species), principally ·OH hydroxyl radicals, are responsible for the large majority of radiation-induced cell death [26,27], both during the “beam ON” time and in mitochondrial damage “ROS waves” at 12 and 144 h after irradiation (Figure 2), it is logical that the new PrC-210 ROS scavenger, a ***direct-acting*** ROS scavenger, not an antioxidant, which outperformed the 13 other most cited antioxidants in PubMed [28] by one to several orders of magnitude, conferred significant protection to all organs in these irradiated mammals (Figure 2 and [22]).

**Figure 12:**
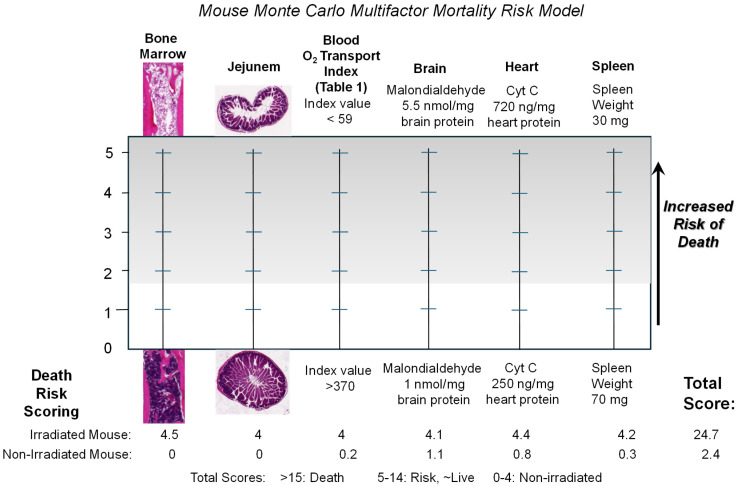
A Monte Carlo multifactor mortality risk model for *non-bacterial sepsis* deaths in irradiated mice. The multiple parallel failure of critical organs in acute radiation syndrome is routinely seen in mice following lethal whole-body irradiation. For the two mice in Figure 4 that showed zero CFUs in their blood cultures at the time of their death, we hypothesize that their deaths are explained by this model of aggregate risk associated with multiple critical organs failing in parallel in lethally irradiated mice. This figure summarizes primary data originally reported in [23], used here with journal permission.

Our first observation of blood-borne *E coli* on day two following the lethal irradiation of mice (Figure 4) was surprising. The exponential expansion of the septic bacteria in the irradiated immune-compromised mice in the following days was less surprising. After studying the earlier histology images from irradiated mice taken from multiple sites along the GI tracts in our earlier study [23], and the significant PrC-210 suppression of the observed GI pathology in mice treated either immediately before or 24 h after lethal irradiation [23], it was clear that breaches in the GI wall integrity in PrC-210-untreated mice (Figure 3B) were common. When we cultured blood from 51 irradiated, near-death mice, we found septic bacteria in 97% of the mice. When we recreated the same IV burden of *E coli* strain in otherwise healthy but immunocompromised mice, it was apparent that the bacterial burden seen in the irradiated mouse bloods, ***alone,*** was 100% lethal to the mice. The observed death of two of 51 mice who had zero blood-borne bacteria, to us, is consistent with a Monte Carlo multifactor mortality risk model (see Figure 12) that we previously offered [23] in which multiple parallel organ failures in irradiated animals, in aggregate, explains death in the 3% of mice with no blood-borne bacteria.

The 97% occurrence of lethal septic bacteria in irradiated outbred mice resulted from two required events, i.e., (i) GI epithelial degradation and the breach of the GI tract wall and (ii) the parallel immune suppression of the mice that we saw which was “required” (see Figure 6A) to enable *lethal* septic infection. It is logical then that a three-drug regimen, in which (i) **PrC-210**, administered orally *once* before or after irradiation, sustains the overall integrity of the entire GI tract (see Figure 3) and helps maintain bone marrow density and recovery time [23]; (ii) **ciprofloxacin**, administered orally daily, here for 14 days, precludes the blood expansion of any leaked GI bacteria until both immune function and GI integrity are restored by 14 days; and (iii) **GCSF**, being administered by subcutaneous injection three times 24–48 h post-irradiation, significantly hastens the restoration of bone marrow cells [23] and immune infection suppression, confers 92% survival benefit to irradiated mice (Figure 11).

**Figure 13:**
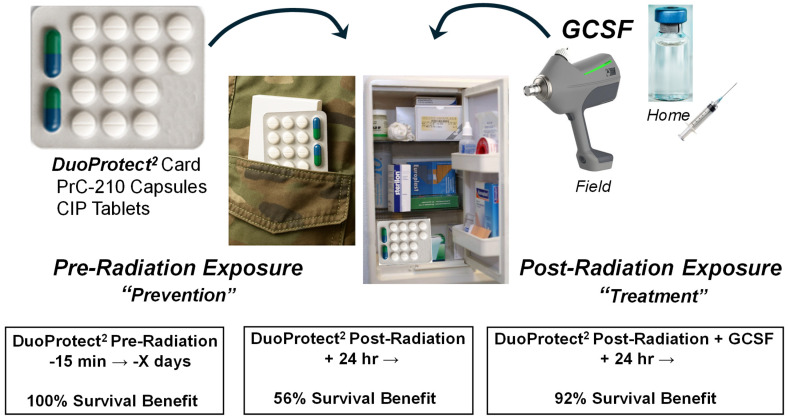
A military and civilian “field-deployed” radiation “prevention” and “treatment” drug regimen. The envisioned DuoProtect^2^ card containing PrC-210 oral gel capsules and 14 ciprofloxacin oral tablets in a water- and oxygen-excluding dose card could be easily deployed in both military and civilian settings. The PrC-210 (alone) oral dose, taken 15–30 min before radiation exposure, was previously shown ([25] and Figure 9) to confer 100% survival against an otherwise 100% lethal dose of whole-body radiation. Daily ciprofloxacin in a post-exposure radiation setting would provide additional supportive protection against bacterial infections. Beginning 24 h after radiation exposure, the oral administration of this field-deployed PrC-210 dose, when combined with the DuoProtect^2^ doses of ciprofloxacin, would confer greater than 55% survival benefit (Figure 10), but when combined with a single SC injection of GCSF (eg., Neulasta, Figure 11), it would confer 92% combined survival benefit from this three-drug regimen. The field deployment of GCSF from any current manufacturer would be challenging but certainly possible for both military and, as importantly, civilian populations, particularly with a 2 year GCSF shelf-life in a family’s home refrigerator.

PrC-210, alone [23,25] or with accompanying ciprofloxacin (Figure 10), conferred 100% survival benefit when taken 60 min before or 56% survival benefit when taken 24 h after lethal irradiation. The addition of GCSF to the PrC-210 + ciprofloxacin DuoProtect^2^^®^ regimen clearly provided a profound additional survival benefit (36%), but the field availability of an injectable drug, stored at 4 °C, is a substantial hurdle to overcome for mass use in a field setting. Figure 13 shows that in addition to, for example, military field use settings, interestingly, the best “field” setting for the three-drug regimen including GCSF would likely be for civilians in their homes. With a 2+-year shelf-life for GCSF when refrigerated, it would be easy for civilians with a prescription to buy the three-drug regimen with a likely 10-year shelf-life for PrC-210 and ciprofloxacin and a replaceable 2-year dose of GCSF, all of which could be administered by oneself, at home. People could self-treat any time that there was a perceived or real threat of nuclear exposure, even for survivors of a thermoblast that were located sufficiently distant from the nuclear event and who then had to deal with fallout and exposures in the hours to days following the nuclear event.

Agent storage and shelf-life for enabling rapid availability to many (millions) at-risk individuals are almost as important as the radioprotective efficacy of the agent. To date, the chemical degradation of crystalline PrC-210 HCl salt has been seen only in settings in which both water and oxygen were present. Monitoring the integrity of PrC-210 thiol is easy; the dissolution of crystals in water or methanol, with immediate analysis by mass spectrometry, yields a single n + 1 peak at 149 atomic mass units [29]. The degradation of thiol to disulfide yields visible peaks at 295 and 148 amu. The storage of PrC-210 HCl crystals in a single bottle, with over 100 openings over a four-year period, showed no detectable disulfide formation when the bottle was flushed with dry N2 prior to its resealing. We formulated PrC-210 HCl crystals in gelatin capsules, and following their oral delivery to mini-swine [30], we observed clear, dose-dependent efficacy as well as clear, dose-dependent toxicity while establishing the mini-swine NOAEL (No Adverse Effect Level) dose, which then provides the easy estimation of oral dosing and likely PrC-210 oral NOAEL doses in humans. The dry formulation of PrC-210 crystals/powder in gel capsules in atmosphere-sealed bottles or plastic foil dose cards would yield easy storage and long-term (years) storage in both at-home medicine chest and field-ready storage settings for mass distribution.

In this thorough analysis of the lethal ARS patho-mechanism and a multidrug regimen to suppress the lethal ARS organ pathology, we found a new and clear explanation of why lethally irradiated mice die from ARS and then identified two drugs which, when administered pre-emptively, confer a 100% survival benefit or 56% if administered after irradiation, as well as a three-drug regimen that confers 92% survival benefit when administered 1–2 days after lethal irradiation. Future studies will pursue the non-human primate corroboration of these results and the transition of PrC-210-based drug regimens to human availability and use.

## 4. Materials and Methods

### 4.1. Materials and PrC-210

PrC-210 HCl (MW: 220) was synthesized for these studies as previously described [31,32]. ICR (CD-1) mice (female, 25–30 gm) were purchased from Envigo (Madison, WI, USA). Neupogen and Neulasta were purchased from Amgen (Thousand Oaks, CA, USA). Caspase 3,7 ApoOne assay kits were purchased from Promega (Fitchburg, WI, USA). Ciprofloxacin, solvents and other chemicals were purchased from Sigma-Aldrich (St Louis, MO, USA). Blood agar plates were from Diamante Scientific (Boardman, OH, USA).

### 4.2. Mouse Studies

All animal experiments complied with international and local animal welfare standards and were approved by the respective regulatory committees. This research was approved by School of Medicine and Public Health Institutional Animal Care and Use Committee at the University of Wisconsin (Protocol #M006610). All procedures were performed in accordance with the Animal Care and Use Policies at the University of Wisconsin. Mice were maintained on a 12 h light/dark cycle and were provided *ad libitum* water and lab chow (Harlan Teklad 8604).

Unanesthetized mice in a plexiglass pie chamber were irradiated in an Xstrahl CIX3 research irradiator (Suwanee, GA, USA) with 300 keV X-rays at 1.37 Gy/min. A total of 30 min before, or 24 h after irradiation, mice received a single systemic dose of PrC-210, either by IP (intraperitoneal) injection, SC (subcutaneous) injection, or an oral gavage delivery route. The delivery route and individual administered doses of PrC-210 are provided in the legends of each figure. As background, the IP MTD (Maximum Tolerated Dose) is 524 mg/kg bw; the SC MTD is 900 mg/kg bw; and the oral MTD, in non-fasted mice, is 2600 mg/kg. The PrC-210 pH 7.2 solution IP injection volume (µls) is 5.6 times the mouse body weight. The PrC-210 pH 7.2 solution SC injection volume (µls) is 1% of the mouse body weight. The PrC-210 pH 4.9 solution (i.e., PrC-210 HCl simply dissolved in water) oral gavage volume is 1% of the mouse body weight. At 24, 30 and 48 h following irradiation, mice received SC injections of 0.17 mg/kg of G-CSF (Neupogen, Amgen, Thousand Oaks, CA, USA). For Neulasta, mice received a single 1 mg/kg bw SC injection 24 h after irradiation.

Following drug treatments, mice were returned to cages and observed twice each day for the next 30 days. In mice euthanized hours to days following irradiation, blood was collected and plasma isolated and frozen at −80 °C.

### 4.3. Mouse Organ Damage Biomarker and Imaging

In studies involving the isolation of mouse organs, mice were euthanized by CO_2_ overdose. A 2 cm segment of the jejunum, 4 cm below the stomach:jejunum juncture, was isolated, and the lumen was flushed with caspase buffer and then homogenized in 1 mL of 4 °C caspase buffer using a Polytron homogenizer (ThermoFisher, Chicago, IL, USA) run at full speed for 30 sec. A total of 500 µL of homogenate was transferred to an Eppendorf tube and frozen on dry ice or liquid nitrogen and stored at −80 °C. In some mice, an adjoining jejunum segment was rinsed in buffer and then immersed in 10% formalin. H&E staining, sample sectioning and mounting, and the microscopy and scanning of 10× images using an Optika Optiscan10 scanner (Optika, Ponteranica, Italy) to generate jpg images were done as described [33]. For the sampling of colon flora microorganisms, a colon segment was sterilely dissected, and its luminal contents were flushed into a sterile tube using 1 mL of sterile PBS (phosphate-buffered saline, pH 7.2). Serial dilutions of the colon flush suspension were plated on blood agar plates.

Activated caspase 3 and 7 activity in mouse jejunum homogenates or plasma was determined using the Apo-ONE fluorescent substrate [23] (Promega, Madison, WI, USA). The activated caspase 3,7 assay was performed as follows: A total of 20 µL of mouse plasma (stored at −80 °C) or buffer dilutions of the jejunum previously homogenized in caspase buffer (50 mM HEPES, pH 7.4, 100 mM NaCl, 1 mM EDTA, 10% glycerol) was mixed with 50 μL of the undiluted Apo-ONE substrate in the wells of a black, opaque 96-well plate to initiate the 60 min reaction; the total reaction volume was 100 µL. Plates were shaken at 175 RPM at 37 °C for 60 min. The fluorescent DEVD caspase 3,7 substrate peptide cleavage product was measured using a BMG Clariostar fluorescent plate reader (BMG Labtech, Ortenberg, Germany) at an excitation wavelength of 499 nm and an emission wavelength of 521 nm. A caspase internal standard was included in each experiment.

For statistical analysis, either Student’s T test was used for simple comparisons between groups, or the Mantel–Cox test was used to compare differences in survi(val between groups. For correlation analyses, Pearson correlation coefficients (“R”) were calculated using Graphpad Prism (Boston, MA, USA).

### 4.4. Mouse Blood Bacterial Analyses

For mouse blood CFU analyses, either healthy or near-death mice following irradiation (i.e., hunched posture, motionless in cage, ragged coat, cold to touch) received a bolus overdose IP injection of pentobarbital (3 mg); when unresponsive, heads were detached by scissors, and blood was collected into sterile tubes. For simple blood CFU screening, 100 µL of blood was spread onto a blood agar plate, and inverted plates were incubated at 37 °C for 24–48 h. Plates were photographed and CFUs recorded. In some cases, bloods were diluted up to 1 million-fold in sterile PBS, and 100 µL of each dilution was plated and CFUs counted to give accurate CFU counts per ml of mouse blood. The mouse blood volume was calculated to be 6.4% of the mouse body weight.

To identify the bacteria that composed the only two colony types ever seen in blood from irradiated mice or a colon flush from a healthy ICR mouse, an example of each colony was picked, expanded in Luria broth, and then subjected to MALDI-TOF mass spectrometry [34] at the Wisconsin State Laboratory of Hygiene (https://www.slh.wisc.edu/) (accessed on 15 August 2025). In studies in which defined numbers of *E. coli* cells were IV-injected into mice, some of the mice were first immunocompromised as described [35,36]. Briefly, in the week before IV *E. coli* injections, mice received two IP injections of 100 mg/kg Cytoxan and two 50 µL IP injections of 1 mg/kg prednisone (0.5 mg/mL in DMSO:water, 1:10). Immediately prior to *E. coli* IV tail vein injections, mice were placed into a plexiglass immobilization holder (Braintree Scientific, Braintree, MA, USA), and tails were immersed for 10 sec in 105 °F tap water. Tail veins were accessed using 30 g needles on 1 cc syringes, and 150 µL of PBS containing defined numbers (0.3–500 million) of *E. coli* cells from titrated overnight cultures was injected as a bolus. Mice were returned to cages and observed for death in the following seven days.

## Data Availability

The original contributions presented in this study are included in the article. Further inquiries can be directed to the corresponding author.
